# Evidence for a Selective Influence of Short-Term Experiences on the Retrieval of Item-Specific Long-Term Bindings

**DOI:** 10.5334/joc.223

**Published:** 2022-05-26

**Authors:** Hannah Dames, Andrea Kiesel, Christina U. Pfeuffer

**Affiliations:** 1 University of Zurich, Department of Psychology, Zurich, Switzerland; 2 Albert-Ludwigs-Universität Freiburg, Department of Psychology, Freiburg, Germany; 3 Albert-Ludwigs-Universität Freiburg, Cognitive Computation Lab, Freiburg, Germany; 4 Catholic University of Eichstätt-Ingolstadt, Department of Psychology, Eichstätt, Germany

**Keywords:** binding, retrieval, memory, action control

## Abstract

Human behavior is guided by prior experience such as bindings between stimuli and responses. Experimentally, this is evident in performance changes when features of the stimulus-response episode reoccur either in the short-term or in the long-term. So far, effects of short-term and long-term bindings are assumed to be independent from one another. In a large-scale re-analysis of eight item-specific stimulus-response priming experiments that orthogonally varied task-specific classifications and actions in the *short-term* (trial N-1 to trial N) and, item-specifically, in the *long-term* (lag of several trials), we tested this independence assumption. In detail, we tested whether short-term experiences (repetitions of classification and action features in two consecutive trials) affected the retrieval of item-specific long-term stimulus-classification (S-C) and stimulus-action (S-A) bindings as well as potential long-term C-A bindings. The retrieval of item-specific long-term S-C bindings (i.e., the size of item-specific S-C priming effects) was affected by the persisting activation of classifications from trial N-1 (short-term priming). There were no further interactions between short-term experiences and long-term bindings. These results suggest a feature-specific, selective influence of short-term priming on long-term binding retrieval (e.g., based on shared feature representations). In contrast, however, we found evidence against an influence of short-term C-A bindings on long-term binding retrieval. This finding suggests that the processes contributing to short-term priming and long-term binding retrieval are dissociable from short-term binding and retrieval processes. Our results thus inform current theories on how short-term and long-term bindings are bound and retrieved (e.g., the BRAC framework).

## Introduction

Both reactive, stimulus-based and proactive, goal-directed human actions strongly rely on prior experience and correspondingly formed bindings (e.g., Frings et al., 2020; Hommel et al., 2001). Features (e.g., stimulus, response, and effect) integrated in these bindings are presumed to be coded in a common representational format (e.g., Hommel et al., 2001). This allows for one feature to retrieve the respective other features bound to it. The Binding and Retrieval in Action Control (BRAC) framework (Frings et al., 2020) incorporates this notion that various features of an event are bound together and later retrieved when one or more of the features re-occur. Thereby, the BRAC framework can account for a variety of phenomena in human action control such as repetition priming, negative priming, and task switching.

Bindings and their effects have been observed in the short-term (e.g., Hommel, 1998, 2004; Giesen et al., 2012; Giesen & Rothermund, 2014; Moeller et al., 2016) as well as in the long-term (e.g., Horner & Henson, 2009; Hsu & Waszak, 2012; Moutsopoulou et al., 2015; Pfeuffer et al., 2017, 2018a, b; Waszak et al., 2003). If and how short-term and long-term bindings interact or rely on shared processes, however, is still a matter of debate. Extending this idea, we tested whether and how short-term experiences affect the retrieval of item-specific long-term bindings.^1^

In the present work, short-term experiences refer to activated features of trial N-1 and/or short-term feature bindings of trial N-1 that may or may not repeat in trial N – potentially, influencing performance in trial N. In contrast, we refer to long-term bindings as associations between features (of stimulus and/or response) formed at least several trials before that may or may not influence performance in trial N when being retrieved. Please note that recent studies have shown that even single-trial priming is sufficient to establish bindings that are retrieved at least several trials later, arguably going beyond a persisting joint representation of features in working memory (e.g., Moutsopoulou et al., 2019, in which bindings were primed twice; Pfeuffer et al., 2017; Whitehead et al., in press). Likewise, Pfeuffer et al. (2017) also argue that long-term binding effects depend upon long-term memory. Hence, we consider the effects of stimuli primed several trials prior to the retrieval context as resulting from long-term bindings.

### Evidence for the binding and retrieval of short-term bindings

Typically, binding phenomena assessed within the BRAC framework are based upon sequential prime-probe paradigms that rely upon the immediate past in trial N-1. That is, studies assessing short-term bindings investigate two consecutive trials. For instance, in stimulus-response binding tasks (S1R1-S2R2 or distractor-response binding), a prime and a probe trial which occur immediately after one another are assessed (e.g., Hommel, 1998, 2004; Giesen et al., 2012; Giesen & Rothermund, 2014; Moeller et al., 2016; see e.g., Frings et al., 2020, for a brief overview). The common assumption in these studies is that stimulus and response features become bound into one episode in trial N-1. In trial N, the entire episode is reactivated if at least one of the features repeats. Evidence for this assumption comes from the typical finding that the repetition of stimulus features between trial N and trial N-1 leads to performance benefits if the same response is required and, conversely, hinders performance if a different response is required (observed for the repetition of both relevant features, e.g., Hommel, 1998, 2004; as well as irrelevant features, e.g., Giesen et al., 2012; Giesen & Rothermund, 2014; Moeller et al., 2016). That is, in trial N, interactions of the stimulus (repetition vs. switch) and response (repetition vs. switch) feature sequence from trial N-1 to trial N are commonly observed. Such findings thus evidence that stimulus and response features become bound into *short-term bindings* which are retrieved in a subsequent trial if at least one of the incorporated features repeats. Importantly, short-term binding effects represent both binding-related processes starting in trial N-1 and retrieval-related processes in trial N (see also Frings et al., 2020). Moreover, it is presently unknown for how long a binding process persists. Hence, disentangling binding and retrieval processes in such paradigms is challenging.

### Evidence for the binding and retrieval of long-term bindings

Of course, human memory extends beyond trial N-1. For instance, item-specific priming studies demonstrate that features associated with certain stimuli can be retrieved at least several trials later even after single-trial item-specific priming (item-specific long-term bindings: e.g., Horner & Henson, 2009; Hsu & Waszak, 2012; Moutsopoulou et al., 2015; Pfeuffer et al., 2017, 2018; Waszak et al., 2003; Whitehead et al., 2020, in press). Specifically, such long-term bindings can be retrieved up to several days later, given at least two prime instances (Moutsopoulou et al., 2019). For instance, Moutsopoulou et al. (2015) had their participants perform one of two classification tasks (size vs. mechanism classification) on pictures of everyday objects by pressing a left or right key (left vs. right action). On each trial, a cue indicated the current classification task and the classification-action mapping such that both the classification task (size vs. mechanism) and the position of the correct response could independently vary on a trial-by-trial basis. Note that based on the size/mechanism of the presented stimulus, the to-be-given classification (size: small/large; mechanism: mechanic/non-mechanic) could thus also independently vary on a trial-by-trial basis. Each stimulus occurred once as a prime and once as a probe, crucially, with a lag of several trials. Moutsopoulou et al. (see also e.g., Pfeuffer et al., 2017, 2018) found that participants responded faster in the probe when the classification task item-specifically repeated (i.e., also the item-specifically required semantic classification, e.g., “small”, repeated) rather than switched (stimulus-classification, S-C, priming effect: i.e., an item-specific long-term binding effect). At the same time, participants responded faster in the probe when the required action (e.g., right key press) item-specifically repeated rather than switched (stimulus-action, S-A, priming effect). These findings evidence the formation (binding in the prime) and later retrieval (in the probe) of long-term bindings (often referred to as associations) between stimulus and response features (i.e., classification and action in this case). Currently, there is no conclusive evidence for an interaction of S-C and S-A retrieval (e.g., S-C-A bindings) even though bindings between classifications and actions (long-term C-A bindings) have been observed in other settings (e.g., Longman et al., 2018).

For such long-term bindings in item-specific priming paradigms, binding and retrieval are separated by several trials and intervening events. Hence, binding and retrieval can be separated as occurring in the prime and probe, respectively. As a consequence, effects of item-specific S-C/S-A mapping repetitions/switches on the probe can only be attributed to retrieval processes. In contrast, for the effects of short-term binding and retrieval (trial N-1 to trial N), binding and retrieval processes cannot be separated as clearly. Either the binding that was formed in trial N-1 simply remains (e.g., because of persisting feature activation between trial N-1 and trial N and/or still ongoing binding processes) and therefore impacts responding in trial N, or the episode of trial N–1 is actually retrieved.

### The impact of short-term experiences on the retrieval of long-term bindings

Short-term and long-term bindings are commonly not assessed in the same experiments. Although some perspectives on memory (e.g., the instance theory of Logan, 1988) appear similar to current ideas of short-term binding (e.g., Frings et al., 2020), several findings point towards the independence of short-term bindings and more long-lasting memory processes (i.e., long-term bindings in our terminology; Colzato et al., 2006; Moeller & Frings, 2017,^2^ but see e.g., Giesen et al., 2020; Schmidt et al., 2020, for findings pointing towards a possible overlap in underlying processes). Nevertheless, we have no reason to assume that the initial representations of stimulus and response features incorporated into short-term and long-term bindings crucially differ. On the contrary, it appears rather inefficient for humans to represent the same features in multiple ways for selective integration in short-term and long-term bindings, respectively. This suggests that – unless a conversion of feature representations is assumed when they are stored more permanently – both short-term and long-term bindings may rely on the same representations of the respective features.

In addition to short-term bindings, feature representations that become activated in trial N-1 may still persist in trial N. For instance, responses are typically faster when the required classification repeats rather than switches between two consecutive trials (e.g., Rogers & Monsell, 1995; Meiran, 1996, for classification repetitions/switches; for an overview on task switching see Kiesel et al., 2010). We refer to these effects as *short-term priming* effects (which – in contrast to short-term bindings – do not require binding or the repetition of another feature; we define *short-term experiences* as an umbrella term encompassing both short-term priming and short-term binding). They suggest that short-term experiences (e.g., a given classification) still affect participants’ behavior in the subsequent trial. Correspondingly, if representations activated in trial N-1 are still active in trial N, they may also influence the retrieval of item-specific long-term bindings. Based on this notion, feature representations (here specifically task-specific semantic classifications and actions) incorporated in both short-term experiences and long-term bindings may interact. That is, when the classification/action in trial N-1 matches the classification/action which has previously been associated with a stimulus presented in trial N (long-term binding), item-specific long-term retrieval effects may be increased as compared to when the classification/action in trial N-1 does not match features of the long-term bindings.

The joint assessment of short-term experiences (activated features in short-term priming and short-term bindings of trial N-1) and long-term binding retrieval in trial N allows for new insights into their relationship that can inform current theorizing regarding the underlying mechanisms of short-term and long-term binding and retrieval. Moreover, as effects of long-term bindings observed in probe trials can unequivocally be associated with retrieval, observed effects can singularly be attributed to interactions with long-term retrieval processes.

### The present study

Here, we investigated the impact of short-term experiences (persisting activation of features as well as short-term bindings of trial N-1) on the retrieval of item-specific long-term bindings in trial N. In addition, we directly tested the assumption that short-term and long-term bindings do not interact as they rely on different processes (Colzato et al., 2006; Moeller & Frings, 2017). To this end, we jointly re-analyzed the data of eight item-specific priming experiments that manipulated the response features classification (task-specific item classification, e.g., “small”) and action (motor output, e.g., right key press). Classifications and actions varied both on a trial-by-trial level between trial N-1 and trial N and on an item-specific level between the prime and probe (trial N; item-specific prime-probe lag of 2–7 trials) of a stimulus (e.g., Pfeuffer et al., 2017, 2018a, b). A joint reanalysis of multiple prior studies on item-specific priming provided the ideal opportunity for addressing our questions with sufficient power.

#### Replication and extension of long-term binding effects

**Figure 1:**
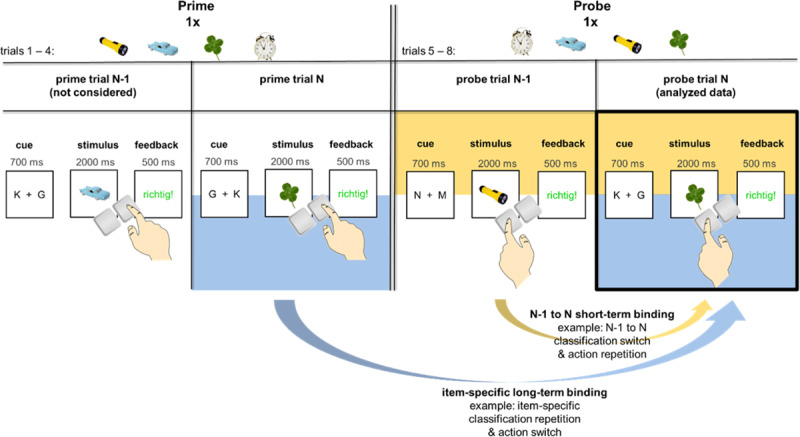
Structure of item-specific priming experiments. *Note*: Participants’ task was to classify stimuli according to their size or mechanism based on a preceding task cue. Stimuli appeared once as a prime and once as a probe (lag 2-7 trials). Between the stimulis’ prime and probe trial their item-specific classification and action mappings could independently repeat or switch allowing us to assess long-term binding effects in the probe trials. By assessing the relation between probe trial N-1 and probe trial N between which the required classification and action could also repeat or switch, we were additionally able to assess short-term experience effects in the probe trials of the same paradigm.

Like in the study of Moutsoupulou et al. (2015), in all re-analyzed experiments, participants performed one of two classification tasks (size vs. mechanism classification) by pressing a left or right key (left vs. right action). As illustrated in Figure 1, the classification-action mappings were indicated by a task-cue prior to stimulus presentation. Stimuli appeared once as a prime and once as a probe (lag 2–7 trials; no direct stimulus repetitions, allowing for the assessment of long-term binding and for a differentiation of binding and retrieval). Both the item-specific classification (size vs. mechanism) and action (left vs. right) mappings could independently repeat or switch between the prime and probe of a stimulus and between trial N-1 and trial N (always stimulus switches). This allowed us to assess long-term binding effects in the probe trials. That is, long-term bindings were operationalized via reaction time (RT) differences in the probe trial (trial N) between item-specific classification repetitions/switches and action repetitions/switches from prime (trial N-2+, lag of several trials) to probe. Like in previous item-specific priming studies (e.g., Moutsoupulou et al., 2015; Pfeuffer et al., 2017, 2018a, b) we expected to observe robust item-specific S-C and S-A retrieval effects (long-term S-C/S-A bindings). We refer to these long-term binding effects as long-term item-specific S-C (short *LT-SC*) binding effects reflecting the differences in RTs between long-term classification repetitions and switches for one specific stimulus (trial N-2+ to trial N). Similarly, participants should generally respond faster in the probe when the required action item-specifically repeats rather than switches. We refer to these differences in RTs between long-term action repetitions and switches as long-term item-specific S-A (*LT-SA*) binding effects. Furthermore, we aimed to test whether classifications and actions might additionally have become bound in the prime (e.g., long-term S-C-A bindings). We reasoned that this large-scale, high-powered re-analysis, in contrast to prior small-scale, individual experiments (e.g., Moutsopoulou et al., 2015; Pfeuffer et al., 2017, 2018a, b), might be able to show interactions of item-specific classification and action bindings in the probe suggesting such long-term bindings between classification and action.

#### Replication of short-term binding effects

Short-term binding effects have been replicated under various circumstances (especially task switching settings are very similar to the design of the present experiments; e.g., Druey & Hübner, 2008, see Kiesel et al., 2010 for a review) and we expected to also observe them in the current reanalysis for the response features classification and action (short-term C-A bindings). Please note that, in the short-term (trial N-1 to trial N) the stimulus always switched. Thus, we did not assess short-term S-C or S-A bindings, but only short-term C-A bindings. We assumed that C and A are bound together in trial N-1, so that the repetition of C in trial N retrieves the previously-executed action. We expected that participants are faster to respond in a current trial N when both or neither classification and/nor action repeated from trial N-1 as compared to when only classification or action repeated (partial repetition costs; see Kiesel et al., 2010 for a review including such effects in task switching; see Frings et al., 2020, for an overview of partial repetition costs in different contexts). Such short-term C-A binding effects should be evident in the interaction between short-term classification (*ST_N-1_C*) sequence (repetition vs. switch) and short-term action (*ST_N-1_A)* sequence (repetition vs. switch).

#### Short-term priming effects – persisting feature activation

Both the classification task (size vs. mechanism judgement)^3^ and the required action (left vs. right key press) could repeat or switch from trial N-1 to trial N. We expected to observe typical short-term priming effects due to persisting activation as observed in typical task-switching experiments (e.g., Rogers & Monsell, 1995; Meiran, 1996; see Kiesel et al., 2010, for a review). That is, participants should be faster to respond in a current trial N when the classification/action repeated from trial N-1 as compared to when it switched. These short-term priming effects reflecting persisting activation of feature representations from N-1 should be evident in longer RTs for short-term classification (*ST_N-1_C*) switches as compared to repetitions as well as longer RTs for short-term action (*ST_N-1_A*) switches as compared to repetitions.

#### Short-term experiences and the retrieval of long-term S-C/S-A bindings

Our key interest were interactions between short-term experiences (short-term priming and short-term binding) and long-term binding retrieval.

##### Short-term priming and the retrieval of long-term S-C/S-A bindings

We hypothesized that both short-term priming and long-term bindings rely on the same classification and action features. Correspondingly, short-term priming (due to persisting activation of classification/action features) could potentially affect the accessibility or strength of specific classification and/or action representations in trial N and thereby reduce/increase corresponding long-term S-C/S-A binding retrieval effects. This would be evident in interactions between *LT-SC* and *ST_N-1_C* and between *LT-SA* and *ST_N-1_A*. If we observed such interactions, it would confirm our hypothesis that features activated based on trial N-1 experiences and features bound within long-term bindings rely on the same representations (e.g., currently activated in working memory). Furthermore, we expected to observe a feature-selective influence of short-term priming on the retrieval of long-term bindings. That is, we assumed an influence of ST_N-1_ C switches/repetitions on the retrieval of long-term S-C but not S-A bindings. Conversely, if we observed that ST_N-1_ C switches/repetitions affected both long-term S-C and S-A binding retrieval, this would indicate that short-term priming generally impeded or facilitated long-term binding retrieval instead.

##### Short-term C-A bindings and the retrieval of long-term S-C/S-A bindings

Furthermore, if short-term bindings and long-term retrieval were not independent, short-term C-A bindings should influence the retrieval of long-term S-C, S-A, and potentially S-C-A bindings. That is, interaction patterns between short-term (ST_N-1_C and ST_N-1_A) and long-term (LT-SC and/or LT-SA) are informative about the interplay of short-term and long-term bindings (i.e., the three-way interactions of LT-SC × ST_N-1_C × ST_N-1_A and of LT-SA × ST_N-1_C × ST_N-1_A as well as the four-way interaction of LT-SC × LT-SA × ST_N-1_C × ST_N-1_A). It is important to highlight that in the present study, the nature of long-term and short-term bindings differs: In the present study, long-term bindings are retrieved when repeating the stimulus between prime and probe. In contrast, short-term C-A bindings are assumed to be retrieved upon repeating at least one of the classification/action features (i.e., the required classification task or the required action) independent of the stimulus. In fact, the stimulus never repeated from trial N-1 to trial N, ruling out additional influences of stimulus repetitions. Here, we investigated whether the activation coming from different retrievals (short-term C-A retrieval and long-term S-C/S-A retrieval) creates additive effects or interacts with one another.

For instance, if action A1 and classification C1 are bound in trial N-1 and classification C1 repeats in trial N, we expected that in trial N, C1 retrieves A1. If this retrieval process activates the same representation that is also activated by the long-term S-A binding retrieval process (e.g., the stimulus in trial N also retrieves the associated action A1), we should observe an interaction between short-term C-A bindings effects and LT-SA. Such interaction patterns would evidence that short-term binding/retrieval processes interacted with the retrieval of long-term bindings, suggesting overlapping feature representations or/and (at least partly) overlapping processes.

## Methods

### Participants

In total, eight experiments assessed in four previously-published studies (39–120 participants each; data previously assessed in Pfeuffer et al., 2017, 2018 a, b, 2020; see Table 1 for details) were included in the reanalysis. These experiments contained the data of 453 participants (mean age = 22.8 years, 348 female, 105 male, 410 right-handed, 43 left-handed). All participants provided written informed consent prior to participation in the original experiments and received course credit or a monetary compensation. In accordance with the data processing of the individual experiments, participants with high error rates (> 30%) or probe trial loss of more than 50% in the reaction time analyses (exclusion of stimuli with incorrect or omitted prime or probe responses) were excluded (see also Pfeuffer et al., 2017, 2018 a, b, 2020, for details on the individual experiments).

**Table 1:** Sample information per prior experiment.


EXPERIMENT	STUDY	N	AGE	GENDER	HANDEDNESS

1	Pfeuffer et al. (2017; Exp. 1)	40	23.0 ± 4.3	10 male 30 female	37 right 3 left

2	Pfeuffer et al. (2017; Exp. 2)	60	23.6 ± 3.8	17 male 43 female	55 right 5 left

3	Pfeuffer et al. (2017; Exp. 3)	39	23.7 ± 3.9	11 male 28 female	37 right 2 left

4	Pfeuffer et al. (2018b)	39	23.1 ± 3.3	9 male 30 female	34 right 5 left

5	Pfeuffer et al. (2020; Exp. 1)	76	19.8 ± 1.8	9 male 67 female	67 right 9 left

6	Pfeuffer et al. (2020; Exp. 2)	120	23.0 ± 4.3	28 male 92 female	108 right 12 left

7	Pfeuffer et al. (2018a; Exp. 2)	40	24.6 ± 4.2	7 male 33 female	36 right 4 left

8	Pfeuffer et al. (2018a; Exp. 1)	39	24.1 ± 4.1	14 male 25 female	36 right 3 left


*Note*: In all experiments, item-specific classification (repetition vs. switch) and item-specific action mappings (repetition vs. switch) between a stimulus’ prime and probe were manipulated. Each stimulus was primed once and probed once with a lag of several (2 to 7) trials – additional conditions were excluded. Data of all participants included in the original papers were selected for this reanalysis. Note that all experiments contained an additional manipulation of prime type (executed vs. verbally coded; manipulated in randomly intermixed blocks). This reanalysis only focused on the executed blocks and trials in which participants actively classified stimuli in prime and probe (verbally coded blocks and trials in which participants merely passively attended to instructions in the prime were discarded).

### Stimuli and apparatus

The stimuli used in the respective experiments originated from the same stimulus set and the apparatus was equivalent. Stimuli were presented on a 24” PC screen in a sound attenuated room. Participants wore headphones and sat approximatively 60 cm away from the display with their index fingers resting on two keys placed left and right in front of them (key distance 13.5 cm).

Everyday objects (presented in picture or word format) had to be classified either according to their size or according to whether they contained a mechanism or not. A reference box (37.5 cm × 30 cm × 13.5 cm) served as reference for the size task. That is, objects smaller than or fitting in the box counted as small, whereas objects larger than or not fitting in the box counted as large. For the mechanism task, objects containing a mechanism (e.g., wheels, levers, hinges, or electronic parts) were to be classified as mechanic, other objects that did not contain any mechanisms were to be classified as non-mechanic. Task cues preceded each object and indicated the current trial’s classification task and the classification-action mapping. When a size classification was required, participants were presented with either the letters “K + G” or “G + K” (first letters of the German words for small – klein and large – groß, respectively). The left/right position of a letter instructed that a left/right response should be performed for the respective classification. For instance, if the task cue was “K + G”, a left key press was to be performed for a small (klein) classification and a right key press for a large (groß) classification. For the mechanism task, the task cues “M + N” or “N + M” appeared (first letters of the German words for mechanic – mechanisch and non-mechanic – nicht-mechanisch, respectively).

Per experiment, 448 to 536 images and 16 to 24 practice images of everyday objects (256 pixels × 256 pixels, ca. 8°; ¼ per classification-action combination) from an initial set by Brady et al. (2008) and Moutsopoulou et al. (2015) and/or the corresponding words (Exp. 7 and 8) describing them were used. Each object was only presented in one block of the experiment, typically once as a prime and once as a probe (other conditions – e.g., a condition in which stimuli were primed multiple times in Pfeuffer et al. (2018) – were not considered for this analysis). Verbal codes were presented via voice recordings of a neutral, female voice (duration 1.8 – 2.3 s, ca. 65 dB) containing an object’s class and an action (e.g., “*klein, rechts*”, Eng.: “small, right”; see Figure 1).

### Design and procedure

All experiments used the same item-specific priming paradigm which manipulated stimulus-classification (S-C) and stimulus-action (S-A) mappings in prime and probe. Each experiment consisted of a large number of short blocks. Per block four new stimuli were first primed and then probed (prime phase: trials 1–4, probe phase: trials 5–8, prime-probe lag: 2–7 trials; diverging conditions were not considered). Per participant, each stimulus only appeared once as a prime and once as a probe in the respective experiment.

In the considered executed blocks, the trial structure of primes and probes was such that the task cue (700 ms) was followed by the stimulus (2000 ms or until response). Once participants responded or 2000 ms had passed, they received feedback regarding the correctness of their responses (“correct!”/”richtig!” in green or “error!”/”Fehler!” in red or “too slow!”/”zu langsam!” in red, 500 ms). Then the next trial ensued. The probe phase immediately followed the prime phase. Participants were instructed to respond fast and accurately.

Between each stimulus’ prime and probe trial, its item-specific classification (LT- SC) and action (LT-SA) mapping could repeat or switch independent from one another. Thus, there were four orthogonal conditions: Item-specific classification repetitions/switches combined with item-specific action repetitions/switches between a stimulus’ prime and probe. Importantly, as the classification task and classification-action mapping expressed in the task cue could switch on a trial-by-trial basis, the classification and action required on trial N-1 and trial N (ST_N-1_A/ST_N-1_C mapping) also varied independent from one another and independent from long-term item-specific action and classification mappings. These independent variations allowed us to assess the interaction of LT-SA/LT-SC mappings and ST_N-1_A/ST_N-1_C mappings.

### Data Analysis

We analyzed log-transformed RTs with Bayesian linear mixed models (BLMMs) using the R *BayesFactor* package (Morey & Rouder, 2015) with the default settings for priors. All categorical predictors were coded as sum-to-zero contrasts. We computed Bayes Factors (BFs) to estimate the strength of evidence for the null and the alternative hypothesis. To this end, we fitted two competing, nested models for each hypothesis: One model that included the factor of interest and one that did not. We then compared the two models to calculate the evidence for/against the null/alternative hypothesis. We considered a BF_10_ larger than 3 as substantial evidence for the alternative hypothesis and a BF_01_ larger than 3 as substantial evidence for the null hypothesis (no effect).

We started model comparisons with the full model that consisted of the fixed factors *LT-SC binding* (long-term classification repetition vs. switch between a stimulus prime and probe), *LT-SA binding* (long-term action repetition vs. switch between a stimulus prime and probe), *ST_N-1_C sequence* (short-term classification repetition vs. switch between trial N-1 and trial N), *ST_N-1_-A sequence* (short-term action repetition vs. switch between trial N-1 and trial N) as well as all corresponding two-way interactions. In addition, it included the three-way *LT-SC binding* × *ST_N-1_C sequence* × *ST_N-1_A sequence* interaction, the three-way *LT-SA binding* × *ST_N-1_C sequence* × *ST_N-1_A sequence* interaction to investigate how short-term bindings (i.e., the interaction of *ST_N-1_C sequence* × *ST_N-1_A sequence)* affected long-term binding retrieval. For the test of the four-way interaction, the model included all possible interactions between the factors. Furthermore, the model included random intercepts for participants and by-participant random slopes for the factors *LT-SC binding, LT-SA binding*, S*T_N-1_C sequence*, and S*T_N-1_A sequence*.

We also added the factor *experiment* (without any interactional terms) to our model to account for variance in RTs caused by the different experimental designs. Comparing the full model that included the factor experiment to the same model without that factor yielded very strong evidence for a difference between experiments (BF_10_ = 6.9 × 10^24^). Therefore, we included this factor in all subsequent model comparisons.

The model comparison procedure was as follows: In a first step, we tested a model including the four-way interaction against a model without that interaction (including all possible three-way interactions). Because we found evidence against this interaction, we did not further consider it. In a second step, we tested the two three-way interactions of interest. Because we found evidence against both three-way interactions, we did not further consider them in any of the subsequent model comparisons. In a second step, we tested all two-way interactions by comparing one model that did not include the interaction of interest to a model including all two-way interactions. In a third step, we tested all main effects. For this, we compared one model that did not include the main effect of interest to a model including all main effects and the interactions for which we found substantial evidence in step 2.

## Results

Data, and analyses scripts are available via the Open Science Framework: https://osf.io/2ky6p/ (Dames et al., 2022).

Our analyses focused on probe trial RTs. We discarded probe trials of stimuli with response omissions or erroneous responses in prime or probe from all analyses (n_trials_ = 22783). We further included only probe trials with correct responses in trial N-1. Finally, we excluded probe trials with RTs above/below 3SD of the individual cell means (n_trial_ = 1002). As in the original studies, we excluded probe trial one and assessed only probe trials two to four to rule out influences of the switch from the prime to the probe phase. Moreover, two types of short-term classification switches between trial N-1 and trial N (*ST_N-1_C*) were possible: First, the classification task could switch between trial N-1 and N which always entailed switches in the required semantic classifications (i.e., from small/large to mechanic/non-mechanic). Second, the classification task could repeat between trial N-1 and trial N, whereas the required semantic classification switched (e.g., from small to large based on the stimuli). These trials did not and could not have a corresponding equivalent of trials with semantic classification repetitions when the classification task switched. Therefore, they could neither be categorized as *ST_N-1_C* repetitions nor switches and were hardly comparable to the other conditions. We therefore excluded all corresponding trials, so that *ST_N-1_C* repetitions always entailed a direct repetition of the classification task and exact semantic classification (e.g., small to small), whereas *ST_N-1_C* switches always entailed a complete switch of the classification task and semantic classification (e.g., small – mechanic).

**Figure 2:**
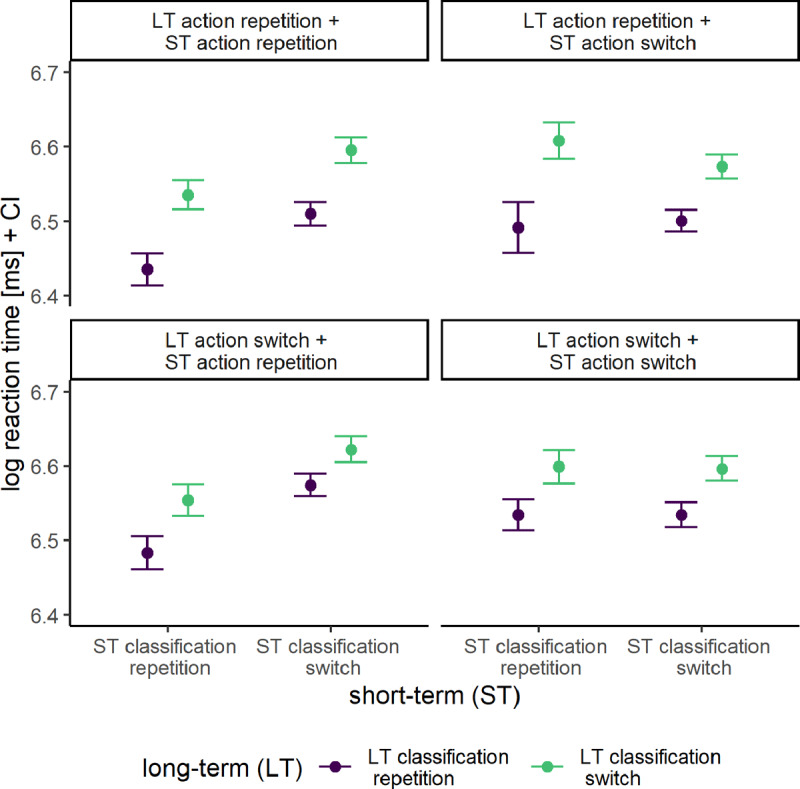
Mean log-transformed Reaction Times Across Short-Term (ST) Sequences and Long-Term (LT) Classification and Action Bindings. *Note*: Error bars represent the within-subject 95% confidence intervals.

Mean log-transformed RTs are shown in Figure 2.

### Replicating long-term binding effects

**Figure 3:**
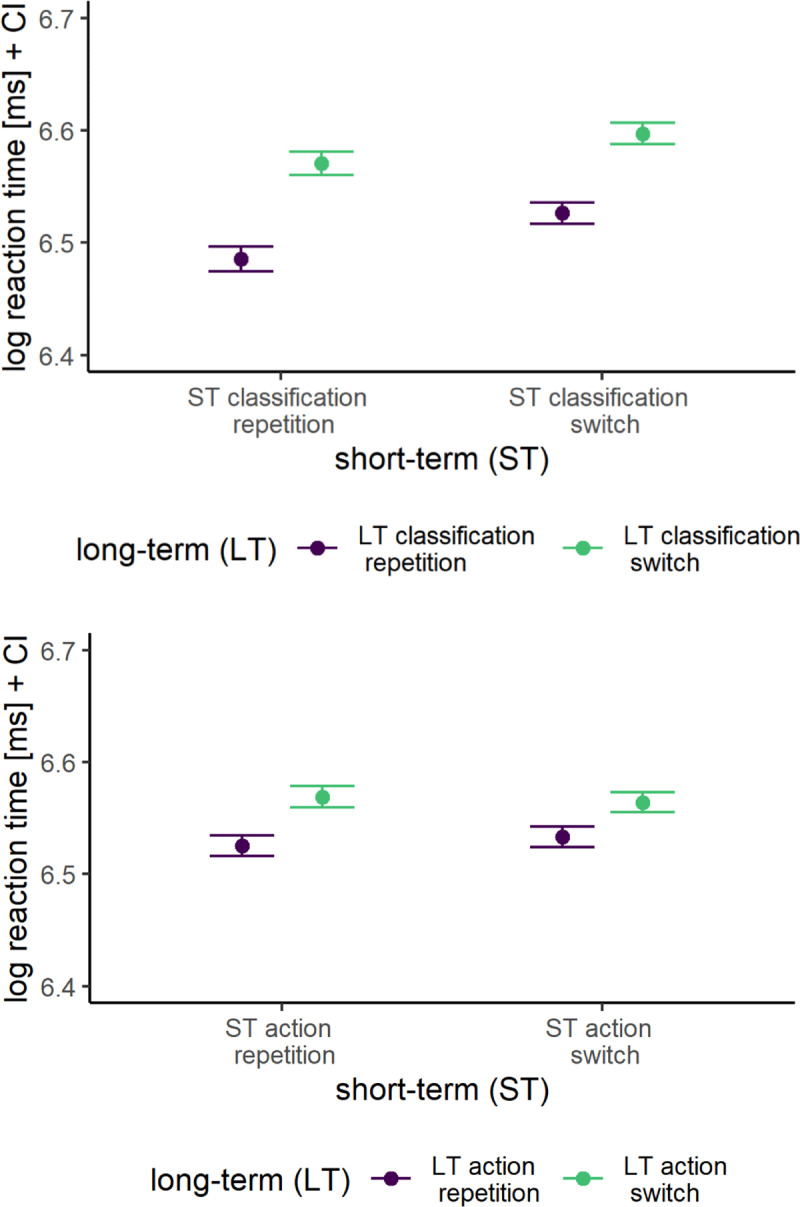
Mean log-transformed Reaction Times Across Short-Term (ST) Sequences and Long-Term (LT) Classification (top) and Action (bottom) Bindings. *Note*: Error bars represent the within-subject 95% confidence intervals.

We found very strong evidence for both LT-SC (BF_10_ = 1.2 × 10^53^) and LT-SA (BF_10_ = 2.3 × 10^8^) binding effects, replicating the individual studies’ long-term binding effects (see Figure 3). That is, participants responded substantially faster when the classification/action item-specifically repeated between prime and probe (trial N-2+ to trial N, lag of several trials) as compared to when it switched. We found no evidence whether the two long-term bindings effects interacted (inconclusive BF_10_ = 1.6).

### Replicating short-term priming and binding effects

**Figure 4:**
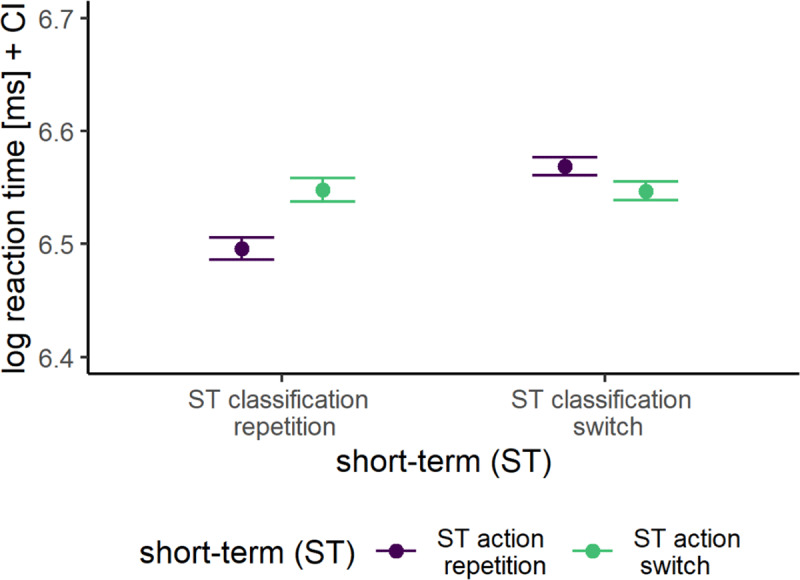
Mean log-transformed Reaction Times Across Short-Term (ST) Classification and Short-Term Action Bindings. *Note*: Error bars represent the within-subject 95% confidence intervals.

Short-term, participants were faster to respond when the classification/action repeated from trial N-1 to the current trial N as evident in substantial ST_N-1_C (BF_10_ = 3.3 × 10^13^) as well as ST_N-1_A (BF_10_ = 1353.0) sequence effects (i.e., evidence for short-term priming effects due to persisting activation of classification/action feature representations; see Figure 4). Moreover, replicating prior short-term C-A binding effects, participants were faster to respond in a current trial N when both classification and action repeated from trial N-1 to trial N as compared to when only classification or action repeated (BF_10_ = 8.1 × 10^28^; i.e., evidence for short-term C-A bindings). Furthermore, switching both classification and action from trial N-1 to trial N resulted in faster responses as compared to when the action repeated but the classification switched (partial repetition costs). However, RTs when switching both classification and action from trial N-1 to trial N were not faster as compared to when the classification repeated but the action switched. This was probably due to generally larger ST_N-1_C than ST_N-1_A effects.

### The influence of short-term priming and short-term bindings on the retrieval of long-term bindings

Importantly, we found evidence for the hypothesis that ST_N-1_C experiences selectively influenced the retrieval of LT-SC bindings, as evident in a substantial interaction between ST_N-1_C sequence and LT-SC bindings (BF_10_ = 24.9; see Figure 3 top). Crucially, ST_N-1_C experiences selectively only influenced the retrieval of LT-SC bindings and did not interact with the retrieval of LT-SA bindings (BF_01_ = 29.4). However, we found no evidence for an interaction between ST_N-1_A sequence and LT-SA bindings (BF_01_ = 2.7; see Figure 3 bottom), but rather indecisive evidence against such an interaction. Likewise, there was no interaction between ST_N-1_A sequence and LT-SC bindings (BF_01_ = 33.1). Further, the retrieval of long-term bindings was not influenced by the interaction between ST_N-1_A and ST_N-1_C experiences (i.e., we found evidence against both three-way interactions LT-SC × ST_N-1_-C × ST_N-1_A, BF_01_ = 59.7, and LT-SA × ST_N-1_C × ST_N-1_A, BF_01_ = 36.0; excluding both interactions against the full model: BF_01_ = 1,853.6). That is, the retrieval of long-term bindings was not influenced by short-term C-A bindings. Likewise, there was strong evidence against a model with the four-way interaction between all factors of interest when comparing a model with that interaction against a model without that interaction (LT-SA × LT-SC × ST_N-1_-C × ST_N-1_A, BF_01_ = 29.4; excluding all three-way interactions as well as the four-way interaction against the full model: BF_01_ = 56,135,988).

In sum, the retrieval of LT-SC bindings was selectively influenced by trial N-1 ST_N-1_C, but not by trial N-1 ST_N-1_A experiences. We found no evidence for the influence of short-term bindings (i.e., the interaction of ST_N-1_C × ST_N-1_A) on long-term binding retrieval.

## Discussion

In this large-scale, high-powered reanalysis, we reassessed the data of eight item-specific priming experiments where unique stimuli appeared once in a prime trial and, several trials later, once in a probe trial. In these experiments task-specific classifications (size vs. mechanism) and associated actions (left vs. right key press) could independently vary in the long-term and in the short-term. *Long-term* bindings were assessed between a stimulus’ single prime (trial N-2+) and probe instance (trial N; lag of several trials; item-specific stimulus repetition). *Short-term* experiences refer to priming and binding effects observed between two consecutive trials (trial N-1 to trial N; only stimulus switches). This allowed us to investigate whether and to what extent short-term experiences interact with the retrieval of long-term bindings. In the present study, the nature of long-term and short-term bindings differed. Whereas long-term bindings were retrieved upon repeating the same stimulus between prime and probe, short-term experiences (short-term classification/action priming and short-term C-A bindings) occurred by repeating at least one classification/action feature (i.e., the required classification task or the required action) irrespective of the stimulus. Here, we investigated whether the activation stemming from different retrievals (short-term C-A retrieval and long-term S-C/S-A retrieval) or priming effects created additive effects or suggested an interaction of long-term and short-term bindings.

Our study replicates past findings demonstrating item-specific long-term bindings (e.g., Horner & Henson, 2009; Hsu & Waszak, 2012; Moutsopoulou et al., 2015; Pfeuffer et al., 2017, 2018a, b; Waszak et al., 2003). As expected, the overall data pattern regarding item-specific long-term S-C/S-A bindings also replicates the findings of the individual studies included in the reanalysis (Pfeuffer 2017, 2018a, 2018b, 2020). Participants responded faster when the required action/classification repeated between prime and probe as compared to when it switched (item-specific long-term S-C/S-A binding). Prior individual studies on long-term bindings did not provide evidence for an interaction between long-term S-C and S-A bindings (i.e., for long-term S-C-A bindings; e.g., Moutsopoulou et al., 2015; Pfeuffer et al., 2017, 2018a, b). Despite increased power due to the large sample size, we found (inconclusive) evidence against an interaction between long-term binding effects of classifications and actions (i.e., S-C-A bindings)– even when controlling for the influence of short-term experiences.

Moreover, we were able to replicate commonly observed short-term priming effects (as observed in prior task-switching studies; e.g., Rogers & Monsell, 1995; Meiran, 1996; see Kiesel et al., 2010 for an overview) and short-term C-A binding effects (e.g., Druey & Hübner, 2008, for the interaction of C-A and Giesen et al., 2012; Giesen & Rothermund, 2014; Hommel, 1998, 2004; Moeller et al., 2016, for other short-term binding effects). That is, participants responded faster when the required classification/action repeated between trial N-1 and trial N than when it switched (short-term priming). Short-term C-A bindings were evident in performance benefits when both or neither the executed classification and/nor action repeated from trial N-1 to trial N as compared to when one of the features switched.

### Short-term priming selectively influences the retrieval of item-specific long-term S-C bindings

Importantly, beyond replicating prior results, our study provides novel findings regarding the relationship of short-term and long-term bindings. Our study is the first to show that the retrieval of long-term bindings interacts with persisting activation of feature representations from short-term priming. Specifically, we found reduced item-specific long-term S-C binding effects when the classification switched between trial N-1 to trial N as compared to when it repeated. This interaction between short-term priming and long-term bindings appears to be feature-selective: Although we found substantive evidence in favor of an interaction of short-term (ST_N-1_C) sequences and item-specific long-term S-C (LT-SC) bindings, there was evidence against an interaction of short-term action sequences (ST_N-1_A) and long-term S-C (LT-SC) bindings (and vice versa). That is, the retrieval of long-term S-C bindings was selectively influenced by whether the classification feature repeated or switched but not by whether the action feature repeated or switched. Conversely, long-term S-A binding retrieval (LT-SA) was not affected by short-term classification sequences (ST_N-1_C).

The results of our Bayesian LMM analysis were inconclusive regarding an interaction of long-term S-C and S-A bindings (inconclusive evidence against such an interaction). However, we observed a selective impact of persisting activation from short-term classification feature representations of trial N-1 (i.e., short-term priming) on item-specific long-term S-C binding effects in trial N. This supports the idea that at least the retrieval of long-term bindings is independent for separate features bound to a stimulus, like classifications and actions.

What processes contributed to this selective interaction between short-term classification sequence (i.e., persisting activation of classification feature representations/short-term priming) and long-term S-C bindings? As influences of the persisting activation of classification feature representations were feature-selective, we can rule out that feature-unselective facilitatory/inhibitory processes contributed to the observed pattern of results. We thus presume that the interaction emerged, because short-term priming and long-term bindings relied on the same feature representations. That is, the persisting activation of the classification feature representation from trial N-1 facilitated the retrieval of item-specific long-term S-C bindings when classifications repeated. That is, the heightened activation of a specific classification feature (e.g., “small”) due to short-term priming could, for instance, have boosted the accessibility or strength of the classification representation and thereby lead to increased item-specific long-term retrieval effects. Conversely or in addition to this process, the simultaneous activation of two different classification features, for instance, “small” in trial N-1 and “mechanic” in trial N, could have interfered with the retrieval of item-specific long-term S-C bindings. That is, the persisting activation of the classification feature representation from trial N-1 hindered the retrieval of item-specific long-term S-C bindings when classifications switched. Such a scenario would be similar to how interference is hypothesized to emerge when features item-specifically switch between the prime and probe of a long-term binding (e.g., Horner & Henson, 2011; Pfeuffer et al., 2020). At present, we can only speculate on the exact details of the underlying mechanisms – that is, whether facilitatory and/or interference processes are at work – and future work is needed to uncover them.

Moreover, we were surprised to observe the selective interaction of short-term priming and long-term bindings only for classifications but not actions. That is, long-term S-A binding retrieval (LT-SA) was not affected by short-term action sequences (ST_N-1_A). Several aspects could have contributed to this dissociation of the features classification and action. First, as classification-action mappings switched on a trial-by-trial basis, the selection of the correct semantic classification necessarily preceded the selection and execution of the action. Thus, the semantic classification was likely the more relevant feature for participants when completing the task. Past research showed that the task relevance of features determines how strongly they are integrated into an episode (see Hommel et al., 2014). Therefore, interactions between persisting action feature representations from trial N-1 and long-term S-A bindings may have been reduced due to the focus on classifications. This idea is supported by the finding that long-term S-C binding effects are typically much larger than long-term S-A binding effects in the item-specific priming paradigm used in all assessed experiments (e.g., Moutsopoulou et al., 2015; Pfeuffer et al., 2017, 2018a, b, 2020). Second, one might also hypothesize that the influence of short-term priming dissipated after affecting classification selection. At present, however, the evidence for/against an interaction of short-term action sequences and long-term S-A bindings is inconclusive and future work is therefore needed to uncover the underlying causes. Such investigations will also be informative regarding the processes underlying differences between features, for instance, due to experimentally-induced relevance. Third, the finding that short-term priming and long-term bindings interacted only for representations of classification features but not for representations of action features could point towards the involvement of different memory sub-systems. For instance, classification features (as declarative representations) could be maintained in declarative working memory and action features (as procedural representations) could be held in procedural working memory (see Oberauer, 2009 for the notion that working memory can be separated into a declarative and a procedural part). However, it has more recently been proposed that procedural and declarative working memory operate in analogous ways (Oberauer et a., 2013). Therefore, the notion that representations of action and classification features are held in different memory sub-systems is still highly speculative and also requires future research.

Finally, we would like to point out that we assessed long-term bindings that were formed several trials prior to the probe trial (lag of 2–7 trials). At this temporal distance, memory traces for bindings were potentially still in a state of higher accessibility when being retrieved two to seven trials later. Bindings probed with a lag of multiple trials between prime and probe have been considered long-term (e.g., Frings et al., 2020) and it has been argued that such bindings, for instance, rely upon long-term rather than working memory (e.g., Pfeuffer et al., 2017). Nevertheless, we encourage future work to study the interaction between short-term experiences and retrieval of long-term bindings probed after longer delays. Such assessments might, for instance, provide additional information regarding the memory systems involved. Likewise introducing a distractor task prior to the probe block may ensure the purging of working memory from any residual memory traces of bindings formed during the prime block. Moreover, studying the impact of multiple prior priming instances on long-term bindings and their interplay with short-term bindings might also be fruitful.

### Long-term binding retrieval does not interact with activation from short-term bindings

Previous work suggested that short-term bindings are independent from (long-term) memory (Colzato et al., 2006; Moeller & Frings, 2017). These studies found that modulating factors influenced short-term and long-term bindings in different ways and thus suggested that short-term and long-term binding processes differ at least in some aspects (Moeller & Frings, 2017). In line with this notion, we found evidence against an interaction of item-specific long-term S-C or S-A binding effects and short-term C-A bindings. This finding suggests that processes contributing to the retrieval of long-term bindings and the binding/retrieval of short-term bindings can be dissociated. Importantly, this was the case although the persisting activation of classification features in trial N-1 affected the retrieval of long-term S-C bindings. Extending prior research, it thus seems that not only the processes underlying the *binding* of short-term and long-term bindings may be different (Colzato et al., 2006; Moeller & Frings, 2017), but also processes underlying the *retrieval* of these bindings. The experimental design of the analyzed experiments allowed us to draw this conclusion, because the binding (prime) and retrieval (probe) of long-term bindings were separated by several intervening events/trials in the assessed item-specific priming experiments. This allowed for a dissociation of binding and retrieval (at least for long-term bindings).

It must be noted, however, that in the present study only long-term but not short-term bindings were retrieved by repeating the same stimulus between prime and probe. Short-term experiences (short-term classification/action priming and short-term C-A bindings) were instead measured upon retrieval of at least one repeating feature (i.e., the required classification task or the required action) irrespective of the stimulus. This means that short-term and long-term bindings in this study differed in terms of their reliance on stimulus representations. Potentially, results may differ when short-term and long-term bindings incorporate the same features including the stimulus. However, future investigations of this question have to bear in mind that a repeated presentation of the stimulus prior to its assessment in the probe might lead to interdependent/integrated long-term and short-term bindings to begin with. That is, prior studies assessing the impact of a multiple instead of a single prior-stimulus presentations on long-term bindings showed increased item-specific retrieval effects (e.g., Moutsopoulou et al., 2015; Pfeuffer et al., 2018a, b).

Short-term and long-term bindings are presumed to be stored in and retrieved from different memory systems, episodic and long-term memory, respectively (e.g., Frings et al., 2020). Our findings provide evidence for this assumption. Here, our observation that, whereas short-term priming interacted with long-term binding retrieval, short-term C-A bindings did not is of special interest. This finding raises the question whether short-term bindings operate on feature representations (or draw on resources) dissociable from those driving short-term priming and long-term binding effects. Our results suggest that C-A bindings do not merely rely upon persisting feature activation from trial N-1 measured in trial N. That is, short-term bindings are dissociable from short-term priming effects (as suggested by the BRAC framework, Frings et al., 2020). Crucially, the present study provides a new methodological approach to further assessing the similarities and differences of short-term priming effects, short-term bindings, and long-term bindings.

## Conclusion

Taken together, our observations strongly suggest that the persisting activation of features from trial N-1 (short-term priming) selectively influences the retrieval of item-specific long-term bindings. We conclude that this interaction emerges due to a reliance on the same feature representations. At the same time, we found evidence against an influence of short-term C-A bindings on the retrieval of item-specific long-term S-C or S-A bindings. This implies that short-term and long-term bindings might not rely on the same feature representations. Furthermore, the observation that only short-term priming but not short-term C-A bindings interacted with long-term binding retrieval has far-reaching consequences. It may suggest that processes contributing to short-term priming are similar to processes guiding our reactions upon long-term binding retrieval (these processes may even operate on the same representations). Conversely, these processes are functional separable from the processing and representations involved in short-term bindings and their effects. This finding informs future theorizing on the interplay of long-term and short-term feature representations and the memory systems involved in their binding and retrieval. Specifically, it further strengthens the view that short-term bindings and memory processes (i.e., long-term bindings) are independent (e.g., Colzato et al., 2006; Moeller & Frings, 2017).

## Data Accessibility Statement

Data, and analyses scripts are available via the Open Science Framework: https://osf.io/2ky6p/ (Dames et al., 2022).

## Footnotes

Please note, that we use the terms “item” and “stimulus” interchangeably to be consistent with previously-established terminology (i.e., “item-specific priming” but “stimulus-based associations”; Horner & Henson, 2009; Hsu & Waszak, 2012; Moutsopoulou et al., 2015; Pfeuffer et al., 2017, 2018; Waszak et al., 2003).

Please note that these studies relied on stimuli primed multiple times, however, as compared to the single-trial priming used in the experiments re-assessed here.

Please note that, for our main analyses, with respect to the classification, we only considered cases with a direct repetition of the classification task and exact semantic classification from trial N-1 to trial N (e.g., small to small) or cases with a complete switch of the classification task and semantic classification between trial N-1 and trial N (e.g., small – mechanic). This was done as only these conditions could item-specifically occur for the long-term mappings. Thus, including only these trials ensured that short-term and long-term conditions were as comparable as possible regarding classification and action sequences.

## References

[1] Colzato, L. S., Raffone, A., & Hommel, B. (2006). What do we learn from binding features? Evidence for multilevel feature integration. Journal of Experimental Psychology: Human Perception and Performance, 32(3), 705. DOI: 10.1037/0096-1523.32.3.705 16822133

[2] Dames, H., Kiesel, A., Ragni, M., & Pfeuffer, C. U. (2022, January). Analysis. Retrieved from osf.io/2ky6p

[3] Druey, M. D., & Hübner, R. (2008). Effects of stimulus features and instruction on response coding, selection, and inhibition: Evidence from repetition effects under task switching. The Quarterly Journal of Experimental Psychology, 61(10), 1573–1600. DOI: 10.1080/17470210701643397 18777444

[4] Frings, C., Hommel, B., Koch, I., Rothermund, K., Dignath, D., Giesen, C., … & Philipp, A. (2020). Binding and retrieval in action control (BRAC). Trends in Cognitive Sciences, 24(5), 375–387. DOI: 10.1016/j.tics.2020.02.004 32298623

[5] Giesen, C., Frings, C., & Rothermund, K. (2012). Differences in the strength of distractor inhibition do not affect distractor–response bindings. Memory & Cognition, 40(3), 373–387. DOI: 10.3758/s13421-011-0157-1 22081277

[6] Giesen, C., & Rothermund, K. (2014). Distractor repetitions retrieve previous responses and previous targets: Experimental dissociations of distractor–response and distractor–target bindings. Journal of Experimental Psychology: Learning, Memory, and Cognition, 40(3), 645. DOI: 10.1037/a0035278 24294915

[7] Giesen, C. G., Schmidt, J. R., & Rothermund, K. (2020). The law of recency: An episodic stimulus-response retrieval account of habit acquisition. Frontiers in Psychology, 10, Article 2927. DOI: 10.3389/fpsyg.2019.02927 32010017 PMC6974578

[8] Hommel, B. (1998). Event files: evidence for automatic integration of stimulus–response episodes. Visual Cognition, 5, 183–216. DOI: 10.1080/713756773

[9] Hommel, B. (2004). Event files: Feature binding in and across perception and action. Trends in cognitive sciences, 8(11), 494–500. DOI: 10.1016/j.tics.2004.08.007 15491903

[10] Hommel, B., Memelink, J., Zmigrod, S., & Colzato, L. S. (2014). Attentional control of the creation and retrieval of stimulus–response bindings. Psychological Research, 78(4), 520–538. DOI: 10.1007/s00426-013-0503-y 23884516

[11] Hommel, B., Müsseler, J., Aschersleben, G., & Prinz, W. (2001). The theory of event coding (TEC): A framework for perception and action planning. Behavioral and brain sciences, 24(5), 849–878. DOI: 10.1017/S0140525X01000103 12239891

[12] Horner, A. J., & Henson, R. N. (2009). Bindings between stimuli and multiple response codes dominate long-lag repetition priming in speeded classification tasks. Journal of Experimental Psychology: Learning, Memory, and Cognition, 35, 757–779. DOI: 10.1037/a0015262 19379048

[13] Hsu, Y. F., & Waszak, F. (2012). Stimulus– classification traces are dominant in response learning. International Journal of Psychophysiology, 86, 262–268. DOI: 10.1016/j.ijpsycho.2012.10.002 23069272

[14] Kiesel, A., Steinhauser, M., Wendt, M., Falkenstein, M., Jost, K., Philipp, A. M., & Koch, I. (2010). Control and interference in task switching—A review. Psychological Bulletin, 136(5), 849–874. DOI: 10.1037/a0019842 20804238

[15] Logan, G. D. (1988). Toward an instance theory of automatization. Psychological Review, 95(4), 492. DOI: 10.1037/0033-295X.95.4.492

[16] Longman, C. S., Milton, F., Wills, A. J., & Verbruggen, F. (2018). Transfer of learned category-response associations is modulated by instruction. Acta Psychologica, 184, 144–167. DOI: 10.1016/j.actpsy.2017.04.004 28454893

[17] Meiran, N. (1996). Reconfiguration of processing mode prior to task performance. Journal of Experimental Psychology: Learning, Memory, and Cognition, 22(6), 1423–1442. DOI: 10.1037/0278-7393.22.6.1423

[18] Moeller, B., & Frings, C. (2017). Dissociation of binding and learning processes. Attention, Perception, & Psychophysics, 79(8), 2590–2605. DOI: 10.3758/s13414-017-1393-7 28752283

[19] Moeller, B., Pfister, R., Kunde, W., & Frings, C. (2016). A common mechanism behind distractor-response and response-effect binding?. Attention, Perception, & Psychophysics, 78(4), 1074–1086. DOI: 10.3758/s13414-016-1063-1 26810573

[20] Morey, R. D., Rouder, J. N., Jamil, T., & Morey, M. R. D. (2015). BayesFactor: Computation of Bayes Factors for Common Designs. Retrieved from https://cran.r-project.org/web/packages/BayesFactor/index.html

[21] Moutsopoulou, K., Pfeuffer, C., Kiesel, A., Yang, Q., & Waszak, F. (2019). How long is long-term priming? Classification and action priming in the scale of days. Quarterly Journal of Experimental Psychology, 72(5), 1183–1199. DOI: 10.1177/1747021818784261 29865936

[22] Moutsopoulou, K., Yang, Q., Desantis, A., & Waszak, F. (2015). Stimulus– classification and stimulus–action associations: Effects of repetition learning and durability. Quarterly Journal of Experimental Psychology: Human Experimental Psychology, 68, 1744–1757. DOI: 10.1080/17470218.2014.984232 25396708

[23] Oberauer, K. (2009). Design for a working memory. Psychology of Learning and Motivation, 51, 45–100. DOI: 10.1016/S0079-7421(09)51002-X

[24] Oberauer, K., Souza, A. S., Druey, M. D., & Gade, M. (2013). Analogous mechanisms of selection and updating in declarative and procedural working memory: Experiments and a computational model. Cognitive Psychology, 66(2), 157–211. DOI: 10.1016/j.cogpsych.2012.11.001 23276689

[25] Pfeuffer, C. U., Hosp, T., Kimmig, E., Moutsopoulou, K., Waszak, F., & Kiesel, A. (2018a). Defining stimulus representation in stimulus–response associations formed on the basis of task execution and verbal codes. Psychological Research, 82(4), 744–758. DOI: 10.1007/s00426-017-0861-y 28391366

[26] Pfeuffer, C. U., Moutsopoulou, K., Pfister, R., Waszak, F., & Kiesel, A. (2017). The power of words: On item-specific stimulus–response associations formed in the absence of action. Journal of Experimental Psychology: Human Perception and Performance, 43(2), 328. DOI: 10.1037/xhp0000317 27831720

[27] Pfeuffer, C. U., Moutsopoulou, K., Waszak, F., & Kiesel, A. (2018b). Multiple priming instances increase the impact of practice-based but not verbal code-based stimulus-response associations. Acta Psychologica, 184, 100–109. DOI: 10.1016/j.actpsy.2017.05.001 28511771

[28] Pfeuffer, C. U., Moutsopoulou, K., Waszak, F., & Kiesel, A. (2020). Execution-based and verbal code-based stimulus–response associations: proportion manipulations reveal conflict adaptation processes in item-specific priming. Psychological Research, 84, 2172–2195. DOI: 10.1007/s00426-019-01220-3 31302777

[29] Rogers, R. D., & Monsell, S. (1995). Costs of a predictible switch between simple cognitive tasks. Journal of Experimental Psychology: General, 124(2), 207–231. DOI: 10.1037/0096-3445.124.2.207

[30] Schmidt, J. R., Giesen, C. G., & Rothermund, K. (2020). Contingency learning as binding? Testing an exemplar view of the colour-word contingency learning effect. The Quarterly Journal of Experimental Psychology, 73(5), 739–761. DOI: 10.1177/1747021820906397 31986984

[31] Waszak, F., Hommel, B., & Allport, A. (2003). Task-switching and longterm priming: Role of episodic stimulus-task bindings in task-shift costs. Cognitive Psychology, 46, 361–413. DOI: 10.1016/S0010-0285(02)00520-0 12809680

[32] Whitehead, P. S., Pfeuffer, C. U., & Egner, T. (in press). Assessing the Durability of One-Shot Stimulus-Control Bindings. Journal of Cognition. 10.5334/joc.218 PMC9400647 36072115

